# (*Z*)-Methyl 3-(2,4-dichloro­phen­yl)-2-[(2-formyl­phen­oxy)meth­yl]acrylate

**DOI:** 10.1107/S1600536811037925

**Published:** 2011-09-30

**Authors:** Rajeswari Gangadharan, K. Sethusankar, Raman Selvakumar, Manickam Bakthadoss

**Affiliations:** a Department of Physics, Ethiraj College for Women (Autonomous), Chennai 600 008, India; b Department of Physics, RKM Vivekananda College (Autonomous), Chennai 600 004, India; c Department of Organic Chemistry, University of Madras, Maraimalai Campus, Chennai 600 025, India

## Abstract

In the title compound, C_18_H_14_Cl_2_O_4_, the mean planes of the methyl acrylate unit and the phenyl ring of the benzaldehyde are approximately orthogonal to each other, making a dihedral angle of 83.31 (6)°. The O atom of the aldehyde group is displaced significantly from the phenyl ring plane by 0.226 (2) Å. The methyl acrylate group adopts an *E* conformation. In the crystal, inversion dimers linked by pairs of C—H⋯O hydrogen bonds generate *R*_2_^2^(24) loops.

## Related literature

For applications of acrylate derivatives, see: De Fraine & Martin (1991 ▶). For a related structure, see: Gong *et al.* (2008 ▶). For *E*-conformation aspects, see: Dunitz & Schweizer (1982 ▶). For resonance effects of acrylate, see: Merlino (1971 ▶); Varghese *et al.* (1986 ▶). For graph-set notation, see: Bernstein *et al.* (1995 ▶).
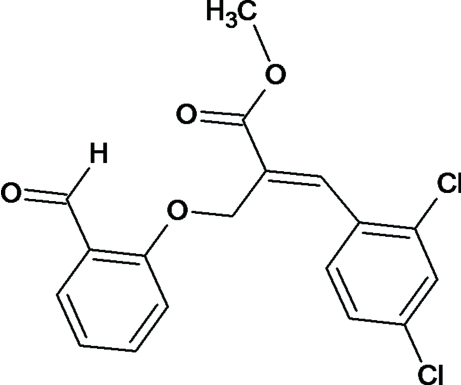


## Experimental

### 

#### Crystal data

C_18_H_14_Cl_2_O_4_
*M* *_r_* = 365.19
Monoclinic, 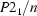

*a* = 17.8151 (7) Å
*b* = 4.9870 (2) Å
*c* = 18.8418 (8) Å
β = 97.834 (2)°
*V* = 1658.36 (12) Å^3^
*Z* = 4
Mo *K*α radiation
μ = 0.41 mm^−1^
*T* = 295 K
0.20 × 0.20 × 0.20 mm

#### Data collection

Bruker Kappa APEXII CCD diffractometer
20245 measured reflections
5036 independent reflections
3310 reflections with *I* > 2σ(*I*)
*R*_int_ = 0.032

#### Refinement

*R*[*F* ^2^ > 2σ(*F* ^2^)] = 0.051
*wR*(*F* ^2^) = 0.149
*S* = 1.08
5036 reflections
218 parameters
H-atom parameters constrained
Δρ_max_ = 0.44 e Å^−3^
Δρ_min_ = −0.31 e Å^−3^

### 

Data collection: *APEX2* (Bruker, 2008 ▶); cell refinement: *SAINT* (Bruker, 2008 ▶); data reduction: *SAINT*; program(s) used to solve structure: *SHELXS97* (Sheldrick, 2008 ▶); program(s) used to refine structure: *SHELXL97* (Sheldrick, 2008 ▶); molecular graphics: *ORTEP-3* (Farrugia, 1997 ▶); software used to prepare material for publication: *SHELXL97* and *PLATON* (Spek, 2009 ▶).

## Supplementary Material

Crystal structure: contains datablock(s) global, I. DOI: 10.1107/S1600536811037925/rk2294sup1.cif

Structure factors: contains datablock(s) I. DOI: 10.1107/S1600536811037925/rk2294Isup2.hkl

Supplementary material file. DOI: 10.1107/S1600536811037925/rk2294Isup3.cml

Additional supplementary materials:  crystallographic information; 3D view; checkCIF report

## Figures and Tables

**Table 1:** Hydrogen-bond geometry (Å, °)

*D*—H⋯*A*	*D*—H	H⋯*A*	*D*⋯*A*	*D*—H⋯*A*
C17—H17⋯O1^i^	0.93	2.49	3.143 (3)	128

Symmetry code: (i) 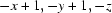.

**Fig. 1.**
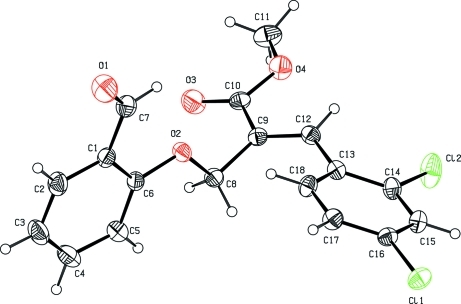
The molecular structure of the title compound with the atom numbering scheme. Displacement ellipsoids are drawn at 30% probability level. H atoms are presented as a small spheres of arbitary radius.

**Fig. 2.**
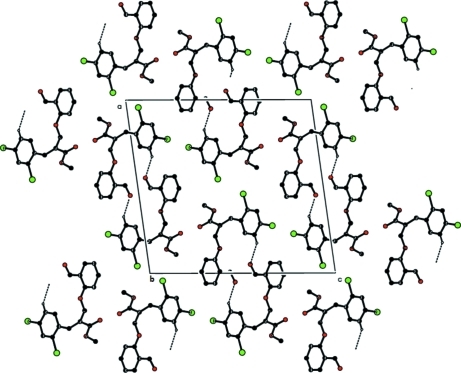
Part of crystal structure of the title compound, showing the formation of R22(24) dimers viewed down b–axis. Dashed lines indicates C—H···O intermolecular interactions with the symmetry code: (i) -x+1, -y+1, -z.

## supplementary crystallographic information

### Comment

Phenyl acrylates and their derivatives are important compounds because of their agrochemical and medical applications (De Fraine & Martin, 1991). The title compound, C_18_H_14_Cl_2_O_4_, consists of a methyl acrylate group, a benzaldehyde group and a dichlorophenyl group as illustrated in (Fig. 1). The acrylate unit is essentially planar with a maximum deviation of -0.017 (2)Å for the C9 atom and forms a dihedral angle of 36.76 (7)° with the phenyl ring (C13–C18). The mean planes formed by the methyl acrylate unit and the phenyl ring (C1–C6) are almost orthogonal to each other, with a dihedral angle of 83.31 (6)°. The interplanar angle between the two phenyl rings (C1–C6) and (C13–C18) is 87.09 (6)°, which shows that they are also almost perpendicular to each other.

The molecules of the title compound display a *Z*–configuration about the C9═C12 double bond. The methyl acrylate moiety adopts an extended *E*–conformation with torsion angles close to 180° as evident from the torsion angles C12–C9–C10–O3 = 177.6 (2)°, C12–C9–C10–O4 = -1.9 (3)°, C9–C10–O4–C11 = 173.79 (19)°, and C8–C9–C10–O4 = -175.07 (18)°. The extended conformation is supported by the fact that the bond angles involving carbonyl O atoms are invariably expanded (Dunitz & Schweizer, 1982). The title compound exhibits structural similarities with the already reported related structure (Gong *et al.*, 2008).

The significant difference in the length of the C10—O4 = 1.332 (3)Å and C11—O4 = 1.438 (3)Å bonds is attributed to a partial contribution from the O^-^–C = O^+^–C resonance structure of the O3═C10—O4—C11 group (Merlino, 1971). This feature, commonly observed in the carboxylic ester group of the substituents in various compounds gives average values of 1.340Å and 1.447Å respectively for these bonds (Varghese *et al.*, 1986).

The crystal packing is stabilized by intermolecular non–classical C—H···O hydrogen bonds with the symmetry code: (i) -*x*+1, -*y*+1, -*z*, which links the molecules into centrosymmetric dimers with graph–set descriptor of R^2^_2_(24) (Bernstein *et al.*, 1995). The packing view of the title compound is shown in Fig. 2.

### Experimental

A solution of salicylaldehyde (3.1 mmol, 0.38 g) and potassium carbonate (3.41 mmol, 0.47 g) in acetonitrile solvent (10 ml) was stirred for 15 minutes at room temperature. To this solution, (*Z*)–methyl–2–(bromomethyl)–3–(2,4–dichlorophenyl)acrylate (3.1 mmol, 1 g) was added dropwise. After the completion of the reaction as indicated by *TLC*, acetonitrile was evaporated. Ethylacetate (15 ml) and water (15 ml) were added to the crude mass and extracted. The organic layer was dried over anhydrous sodium sulfate. Removal of solvent led to the crude product which was purified through pad of silica gel (100–200 mesh) using ethylacetate and hexanes (1:9) as solvents. The pure title compound was obtained as a colourless solid (1 g, 89%). Recrystallization was carried out using ethylacetate as solvent.

### Refinement

The hydrogen atoms were placed in calculated positions with C—H = 0.93Å to 0.97Å and refined in the riding model with fixed isotropic displacement parameters: *U*_iso_(H) = 1.5*U*_eq_(C) for methyl group and *U*_iso_(H) = 1.2*U*_eq_(C) for other groups.

### Crystal data

**Table:**

C_18_H_14_Cl_2_O_4_	*F*(000) = 752
*M**_r_* = 365.19	*D*_x_ = 1.463 Mg m^−^^3^
Monoclinic, *P*2_1_/*n*	Mo *K*α radiation, λ = 0.71073 Å
Hall symbol: -P 2yn	Cell parameters from 5036 reflections
*a* = 17.8151 (7) Å	θ = 1.5–30.6°
*b* = 4.9870 (2) Å	µ = 0.41 mm^−^^1^
*c* = 18.8418 (8) Å	*T* = 295 K
β = 97.834 (2)°	Block, colourless
*V* = 1658.36 (12) Å^3^	0.20 × 0.20 × 0.20 mm
*Z* = 4	

### Data collection

**Table:**

Bruker Kappa APEXII CCD diffractometer	3310 reflections with *I* > 2σ(*I*)
Radiation source: fine–focus sealed tube	*R*_int_ = 0.032
graphite	θ_max_ = 30.6°, θ_min_ = 1.5°
ω and φ scans	*h* = −25→25
20245 measured reflections	*k* = −7→3
5036 independent reflections	*l* = −26→26

### Refinement

**Table:**

Refinement on *F*^2^	Primary atom site location: structure-invariant direct methods
Least-squares matrix: full	Secondary atom site location: difference Fourier map
*R*[*F*^2^ > 2σ(*F*^2^)] = 0.051	Hydrogen site location: inferred from neighbouring sites
*wR*(*F*^2^) = 0.149	H-atom parameters constrained
*S* = 1.08	*w* = 1/[σ^2^(*F*_o_^2^) + (0.0511*P*)^2^ + 1.2391*P*] where *P* = (*F*_o_^2^ + 2*F*_c_^2^)/3
5036 reflections	(Δ/σ)_max_ = 0.001
218 parameters	Δρ_max_ = 0.44 e Å^−^^3^
0 restraints	Δρ_min_ = −0.31 e Å^−^^3^

### Special details

**Table:**

Geometry. All s.u.'s (except the s.u. in the dihedral angle between two l.s. planes) are estimated using the full covariance matrix. The cell s.u.'s are taken into account individually in the estimation of s.u.'s in distances, angles and torsion angles; correlations between s.u.'s in cell parameters are only used when they are defined by crystal symmetry. An approximate (isotropic) treatment of cell s.u.'s is used for estimating s.u.'s involving l.s. planes.
Refinement. Refinement of *F*^2^ against ALL reflections. The weighted *R*–factor *wR* and goodness of fit *S* are based on *F*^2^, conventional *R*–factors *R* are based on *F*, with *F* set to zero for negative *F*^2^. The threshold expression of *F*^2^ > σ(*F*^2^) is used only for calculating *R*–factors(gt) *etc*. and is not relevant to the choice of reflections for refinement. *R*–factors based on *F*^2^ are statistically about twice as large as those based on *F*, and *R*–factors based on ALL data will be even larger.

### Fractional atomic coordinates and isotropic or equivalent isotropic displacement parameters (Å^2^)

**Table:**

	*x*	*y*	*z*	*U*_iso_*/*U*_eq_	
C1	0.50976 (11)	0.5072 (5)	−0.12201 (11)	0.0361 (5)	
C2	0.44223 (13)	0.4038 (6)	−0.15665 (13)	0.0485 (6)	
H2	0.3966	0.4732	−0.1461	0.058*	
C3	0.44138 (15)	0.2022 (6)	−0.20587 (15)	0.0570 (7)	
H3	0.3957	0.1370	−0.2293	0.068*	
C4	0.50939 (15)	0.0966 (6)	−0.22037 (13)	0.0535 (7)	
H4	0.5091	−0.0430	−0.2532	0.064*	
C5	0.57791 (13)	0.1942 (5)	−0.18709 (12)	0.0433 (5)	
H5	0.6233	0.1199	−0.1968	0.052*	
C6	0.57799 (11)	0.4045 (5)	−0.13900 (10)	0.0330 (4)	
C7	0.50730 (12)	0.7188 (5)	−0.06754 (13)	0.0448 (5)	
H7	0.5525	0.7996	−0.0480	0.054*	
C8	0.71317 (11)	0.4083 (5)	−0.11953 (11)	0.0352 (5)	
H8A	0.7138	0.3953	−0.1708	0.042*	
H8B	0.7190	0.2295	−0.0993	0.042*	
C9	0.77651 (11)	0.5838 (4)	−0.08667 (10)	0.0320 (4)	
C10	0.80027 (12)	0.7915 (5)	−0.13589 (11)	0.0363 (5)	
C11	0.89107 (15)	1.1174 (6)	−0.15499 (15)	0.0550 (7)	
H11A	0.9213	1.0211	−0.1849	0.083*	
H11B	0.9219	1.2478	−0.1272	0.083*	
H11C	0.8503	1.2065	−0.1844	0.083*	
C12	0.81596 (11)	0.5482 (5)	−0.02196 (10)	0.0341 (4)	
H12	0.8557	0.6672	−0.0090	0.041*	
C13	0.80390 (11)	0.3428 (5)	0.03113 (10)	0.0336 (4)	
C14	0.86531 (11)	0.2247 (5)	0.07368 (11)	0.0385 (5)	
C15	0.85715 (12)	0.0298 (5)	0.12376 (11)	0.0406 (5)	
H15	0.8992	−0.0465	0.1510	0.049*	
C16	0.78493 (12)	−0.0490 (5)	0.13245 (11)	0.0355 (4)	
C17	0.72198 (12)	0.0627 (5)	0.09273 (12)	0.0396 (5)	
H17	0.6736	0.0076	0.0994	0.048*	
C18	0.73194 (11)	0.2575 (5)	0.04289 (11)	0.0379 (5)	
H18	0.6895	0.3343	0.0163	0.046*	
O1	0.44948 (10)	0.7910 (5)	−0.04748 (11)	0.0678 (6)	
O2	0.64275 (7)	0.5222 (3)	−0.10540 (8)	0.0383 (4)	
O3	0.76863 (12)	0.8266 (4)	−0.19514 (9)	0.0619 (5)	
O4	0.86052 (9)	0.9328 (4)	−0.10778 (9)	0.0506 (4)	
Cl1	0.77343 (4)	−0.29394 (13)	0.19535 (3)	0.04866 (17)	
Cl2	0.95702 (3)	0.3180 (2)	0.06295 (4)	0.0730 (3)	

### Atomic displacement parameters (Å^2^)

**Table:**

	*U*^11^	*U*^22^	*U*^33^	*U*^12^	*U*^13^	*U*^23^
C1	0.0322 (9)	0.0358 (12)	0.0396 (10)	0.0000 (9)	0.0021 (8)	0.0067 (9)
C2	0.0342 (11)	0.0538 (16)	0.0554 (14)	−0.0046 (11)	−0.0011 (9)	0.0064 (12)
C3	0.0452 (13)	0.0623 (19)	0.0596 (15)	−0.0194 (13)	−0.0069 (11)	−0.0025 (14)
C4	0.0615 (16)	0.0502 (17)	0.0471 (13)	−0.0177 (13)	0.0015 (11)	−0.0084 (12)
C5	0.0444 (12)	0.0437 (14)	0.0416 (11)	−0.0037 (10)	0.0055 (9)	−0.0036 (10)
C6	0.0311 (9)	0.0340 (12)	0.0329 (9)	−0.0020 (8)	0.0007 (7)	0.0038 (8)
C7	0.0311 (10)	0.0473 (15)	0.0557 (13)	0.0040 (10)	0.0045 (9)	−0.0018 (11)
C8	0.0306 (9)	0.0370 (12)	0.0381 (10)	0.0034 (8)	0.0058 (8)	−0.0056 (9)
C9	0.0297 (9)	0.0323 (11)	0.0355 (9)	0.0038 (8)	0.0095 (7)	−0.0019 (8)
C10	0.0395 (10)	0.0320 (12)	0.0390 (10)	0.0048 (9)	0.0113 (8)	−0.0029 (9)
C11	0.0554 (15)	0.0443 (16)	0.0707 (17)	−0.0052 (12)	0.0279 (13)	0.0070 (13)
C12	0.0302 (9)	0.0358 (12)	0.0370 (10)	−0.0040 (8)	0.0067 (7)	−0.0030 (9)
C13	0.0321 (9)	0.0367 (13)	0.0321 (9)	−0.0015 (8)	0.0052 (7)	−0.0017 (8)
C14	0.0274 (9)	0.0489 (14)	0.0390 (10)	−0.0018 (9)	0.0038 (8)	0.0005 (10)
C15	0.0346 (10)	0.0477 (15)	0.0382 (11)	0.0044 (10)	0.0008 (8)	0.0040 (10)
C16	0.0402 (10)	0.0320 (12)	0.0348 (9)	0.0008 (9)	0.0073 (8)	0.0011 (8)
C17	0.0306 (9)	0.0428 (14)	0.0466 (11)	−0.0009 (9)	0.0095 (8)	0.0032 (10)
C18	0.0288 (9)	0.0443 (14)	0.0410 (10)	0.0051 (9)	0.0058 (8)	0.0053 (9)
O1	0.0378 (9)	0.0831 (16)	0.0839 (14)	0.0108 (10)	0.0135 (9)	−0.0210 (12)
O2	0.0259 (6)	0.0416 (9)	0.0468 (8)	0.0029 (6)	0.0022 (6)	−0.0116 (7)
O3	0.0852 (14)	0.0552 (13)	0.0422 (9)	−0.0142 (10)	−0.0025 (9)	0.0084 (9)
O4	0.0436 (9)	0.0537 (12)	0.0549 (10)	−0.0114 (8)	0.0081 (7)	0.0109 (8)
Cl1	0.0563 (3)	0.0425 (4)	0.0483 (3)	0.0043 (3)	0.0113 (2)	0.0107 (3)
Cl2	0.0285 (3)	0.1081 (7)	0.0808 (5)	−0.0100 (3)	0.0017 (3)	0.0344 (5)

### Geometric parameters (Å, °)

**Table:**

C1—C2	1.387 (3)	C10—O3	1.193 (3)
C1—C6	1.396 (3)	C10—O4	1.332 (3)
C1—C7	1.477 (3)	C11—O4	1.438 (3)
C2—C3	1.367 (4)	C11—H11A	0.9600
C2—H2	0.9300	C11—H11B	0.9600
C3—C4	1.382 (4)	C11—H11C	0.9600
C3—H3	0.9300	C12—C13	1.468 (3)
C4—C5	1.383 (3)	C12—H12	0.9300
C4—H4	0.9300	C13—C14	1.396 (3)
C5—C6	1.386 (3)	C13—C18	1.397 (3)
C5—H5	0.9300	C14—C15	1.376 (3)
C6—O2	1.370 (2)	C14—Cl2	1.737 (2)
C7—O1	1.200 (3)	C15—C16	1.376 (3)
C7—H7	0.9300	C15—H15	0.9300
C8—O2	1.435 (2)	C16—C17	1.378 (3)
C8—C9	1.494 (3)	C16—Cl1	1.733 (2)
C8—H8A	0.9700	C17—C18	1.379 (3)
C8—H8B	0.9700	C17—H17	0.9300
C9—C12	1.334 (3)	C18—H18	0.9300
C9—C10	1.490 (3)		
			
C2—C1—C6	118.8 (2)	O3—C10—C9	123.1 (2)
C2—C1—C7	119.1 (2)	O4—C10—C9	113.71 (18)
C6—C1—C7	122.06 (19)	O4—C11—H11A	109.5
C3—C2—C1	121.4 (2)	O4—C11—H11B	109.5
C3—C2—H2	119.3	H11A—C11—H11B	109.5
C1—C2—H2	119.3	O4—C11—H11C	109.5
C2—C3—C4	119.0 (2)	H11A—C11—H11C	109.5
C2—C3—H3	120.5	H11B—C11—H11C	109.5
C4—C3—H3	120.5	C9—C12—C13	127.44 (19)
C3—C4—C5	121.3 (3)	C9—C12—H12	116.3
C3—C4—H4	119.3	C13—C12—H12	116.3
C5—C4—H4	119.3	C14—C13—C18	116.4 (2)
C4—C5—C6	119.0 (2)	C14—C13—C12	120.66 (18)
C4—C5—H5	120.5	C18—C13—C12	122.91 (18)
C6—C5—H5	120.5	C15—C14—C13	122.98 (19)
O2—C6—C5	123.51 (19)	C15—C14—Cl2	117.26 (16)
O2—C6—C1	116.20 (19)	C13—C14—Cl2	119.75 (17)
C5—C6—C1	120.28 (19)	C14—C15—C16	118.11 (19)
O1—C7—C1	122.8 (2)	C14—C15—H15	120.9
O1—C7—H7	118.6	C16—C15—H15	120.9
C1—C7—H7	118.6	C15—C16—C17	121.6 (2)
O2—C8—C9	108.75 (17)	C15—C16—Cl1	118.83 (17)
O2—C8—H8A	109.9	C17—C16—Cl1	119.53 (17)
C9—C8—H8A	109.9	C16—C17—C18	118.94 (19)
O2—C8—H8B	109.9	C16—C17—H17	120.5
C9—C8—H8B	109.9	C18—C17—H17	120.5
H8A—C8—H8B	108.3	C17—C18—C13	121.90 (19)
C12—C9—C10	120.18 (19)	C17—C18—H18	119.0
C12—C9—C8	125.1 (2)	C13—C18—H18	119.0
C10—C9—C8	114.33 (17)	C6—O2—C8	116.56 (16)
O3—C10—O4	123.2 (2)	C10—O4—C11	116.43 (19)
			
C6—C1—C2—C3	1.0 (4)	C9—C12—C13—C14	143.0 (2)
C7—C1—C2—C3	−178.0 (2)	C9—C12—C13—C18	−38.0 (3)
C1—C2—C3—C4	1.1 (4)	C18—C13—C14—C15	1.5 (3)
C2—C3—C4—C5	−1.2 (4)	C12—C13—C14—C15	−179.4 (2)
C3—C4—C5—C6	−0.8 (4)	C18—C13—C14—Cl2	−179.99 (17)
C4—C5—C6—O2	−177.4 (2)	C12—C13—C14—Cl2	−0.9 (3)
C4—C5—C6—C1	3.0 (3)	C13—C14—C15—C16	−0.7 (4)
C2—C1—C6—O2	177.3 (2)	Cl2—C14—C15—C16	−179.27 (18)
C7—C1—C6—O2	−3.8 (3)	C14—C15—C16—C17	−0.2 (3)
C2—C1—C6—C5	−3.1 (3)	C14—C15—C16—Cl1	−179.91 (18)
C7—C1—C6—C5	175.9 (2)	C15—C16—C17—C18	0.3 (4)
C2—C1—C7—O1	6.2 (4)	Cl1—C16—C17—C18	179.96 (17)
C6—C1—C7—O1	−172.7 (2)	C16—C17—C18—C13	0.6 (3)
O2—C8—C9—C12	94.7 (2)	C14—C13—C18—C17	−1.4 (3)
O2—C8—C9—C10	−92.5 (2)	C12—C13—C18—C17	179.5 (2)
C12—C9—C10—O3	177.6 (2)	C5—C6—O2—C8	−3.0 (3)
C8—C9—C10—O3	4.4 (3)	C1—C6—O2—C8	176.68 (18)
C12—C9—C10—O4	−1.9 (3)	C9—C8—O2—C6	172.41 (17)
C8—C9—C10—O4	−175.07 (18)	O3—C10—O4—C11	−5.7 (3)
C10—C9—C12—C13	−175.53 (19)	C9—C10—O4—C11	173.79 (19)
C8—C9—C12—C13	−3.1 (3)		

### Hydrogen-bond geometry (Å, °)

**Table:**

*D*—H···*A*	*D*—H	H···*A*	*D*···*A*	*D*—H···*A*
C17—H17···O1^i^	0.93	2.49	3.143 (3)	128

Symmetry codes: (i) −*x*+1, −*y*+1, −*z*.

## References

[bb1] Bernstein, J., Davis, R. E., Shimoni, L. & Chang, N.-L. (1995). *Angew. Chem. Int. Ed. Engl.* **34**, 1555–1573.

[bb2] Bruker (2008). *APEX2* and *SAINT* Bruker AXS Inc., Madison, Wisconsin, USA.

[bb3] De Fraine, P. J. & Martin, A. (1991). US Patent 5 055 471.

[bb4] Dunitz, J. D. & Schweizer, B. W. (1982). *Helv. Chim. Acta*, **65**, 1547–1554.

[bb5] Farrugia, L. J. (1997). *J. Appl. Cryst.* **30**, 565.

[bb6] Gong, H.-B., Wang, J., Liu, Y. & Wang, L. (2008). *Acta Cryst.* E**64**, o2373. 10.1107/S1600536808037318 PMC2959995 21581345

[bb7] Merlino, S. (1971). *Acta Cryst.* B**27**, 2491–2492.

[bb8] Sheldrick, G. M. (2008). *Acta Cryst.* A**64**, 112–122. 10.1107/S0108767307043930 18156677

[bb9] Spek, A. L. (2009). *Acta Cryst.* D**65**, 148–155. 10.1107/S090744490804362X PMC2631630 19171970

[bb10] Varghese, B., Srinivasan, S., Padmanabhan, P. V. & Ramadas, S. R. (1986). *Acta Cryst.* C**42**, 1544–1546.

